# Synthesis of NVCL-NIPAM Hydrogels Using PEGDMA as a Chemical Crosslinker for Controlled Swelling Behaviours in Potential Shapeshifting Applications

**DOI:** 10.3390/gels9030248

**Published:** 2023-03-20

**Authors:** Billy Shu Hieng Tie, Elaine Halligan, Shuo Zhuo, Gavin Keane, Luke Geever

**Affiliations:** 1 Polymer, Recycling, Industrial, Sustainability and Manufacturing (PRISM) Centre, Technological University of the Shannon: Midlands Midwest, N37 HD68 Athlone, Ireland; 2 Centre for Industrial Service & Design, Technological University of the Shannon: Midlands Midwest, N37 HD68 Athlone, Ireland; 3 Applied Polymer Technologies Gateway, Materials Research Institute, Technological University of the Shannon: Midlands Midwest, N37 HD68 Athlone, Ireland

**Keywords:** hydrogels, soft materials, smart materials, biomaterials, hydrophilic, temperature-responsive, lower critical solution temperature, photopolymerisation, chemically crosslinking, copolymers

## Abstract

Stimuli-responsive hydrogels have recently gained interest within shapeshifting applications due to their capabilities to expand in water and their altering swelling properties when triggered by stimuli, such as pH and heat. While conventional hydrogels lose their mechanical strength during swelling, most shapeshifting applications require materials to have mechanical strength within a satisfactory range to perform specified tasks. Thus, stronger hydrogels are needed for shapeshifting applications. Poly (N-isopropylacrylamide) (PNIPAm) and poly (N-vinyl caprolactam) (PNVCL) are the most popular thermosensitive hydrogels studied. Their close-to-physiological lower critical solution temperature (LCST) makes them superior candidates in biomedicine. In this study, copolymers made of NVCL and NIPAm and chemically crosslinked using poly (ethylene glycol) dimethacrylate (PEGDMA) were fabricated. Successful polymerisation was proven via Fourier transform infrared spectroscopy (FTIR). The effects of incorporating comonomer and crosslinker on the LCST were found minimal using cloud-point measurements, ultraviolet (UV) spectroscopy, and differential scanning calorimetry (DSC). Formulations that completed three cycles of thermo-reversing pulsatile swelling are demonstrated. Lastly, rheological analysis validated the mechanical strength of PNVCL, which was improved due to the incorporation of NIPAm and PEGDMA. This study showcases potential smart thermosensitive NVCL-based copolymers that can be applied in the biomedical shapeshifting area.

## 1. Introduction

Hydrogels are three-dimensional hydrophilic polymeric networks that can absorb large amounts of water into their polymeric matrix [1]. Depending on the types of crosslinking, chemically crosslinked hydrogels are stable, whereas physically crosslinked hydrogels disintegrate and dissolve sooner or later [2]. Chemically crosslinked hydrogels are also known as permanent or chemical gels. Their molecular chains are connected by covalent bonds; while physically crosslinked hydrogels are known as reversible or physical gels, and their polymeric network is formed by molecular entanglements and often with secondary forces, such as ionic, hydrogen bonding, and hydrophobic forces [3]. 

In fact, hydrogels were the first biomaterials created for applying in the human body, they were used as filling after enucleation of the eye [4,5]. There are many benefits for utilising hydrogels as biomaterials because of their biodegradability, biocompatibility, hydrophilicity, superabsorbancy, viscoelasticity, softness, and fluffiness [6]. To date, hydrogels have been applied in applications, such as contact lenses [7], tissue engineering [8,9,10], drug delivery [11,12,13,14,15], wound dressings [16], biosensors [17], and hygiene products [3,18].

Starting in the 1970s, stimuli-responsive hydrogels, also known as smart hydrogels that react to changes in the external environment, were studied [19]. These smart hydrogels can react based on a change in the environmental conditions, including pH, temperature, ionic strength, solvent type, electric and magnetic fields, light, and enzyme concentration. The response and magnitude of response of the stimuli-responsive hydrogels to stimuli are decided by the nature of their monomer, charge density, pendant chains, and the degree of crosslinking [1]. 

While multi-stimuli-responsive hydrogels currently exist [20], when it comes to research, single-stimulus-responsive hydrogels are generally more studied. One of the most popular groups of stimuli-responsive hydrogels is thermosensitive hydrogels. Thermosensitive hydrogels are smart hydrogels that alter their swelling behaviours due to changes in temperature, and they can be classified into positive thermosensitive and negative thermosensitive hydrogels [12]. 

Negative thermosensitive polymers are soluble at temperatures lower than their lower critical solution temperature (LCST) but are insoluble above their LCST; while positive thermosensitive polymers are insoluble at temperatures lower than their upper critical solution temperature (UCST) but are soluble above their UCST [21]. This smart feature of thermosensitive hydrogels gives them the ability to exhibit two different states above and below their critical solution temperatures and creates a wide range of applications for these hydrogels to be potentially applied. 

One example where thermosensitive hydrogels are being applied is in the drug delivery field, where the drug content is released when the environmental temperature is above their LCST [22]. However, in shapeshifting applications, this could mean that thermosensitive smart hydrogels can exhibit two different shapes when the environmental temperature is above or below their critical solution temperature.

Van Manen et al. (2018) reviewed the shapeshifting mechanisms of hydrogels [23], and it seems that hydrogels should be studied more in the shapeshifting area as compared with other shapeshifting materials, such as shape memory polymers [24], as hydrogels are the most fitting shapeshift-able materials to be applied in the biomedical area owing to their characteristics. 

The term ‘4D printing’ emerged in 2013 [25], where a time factor was introduced to the 3D-printed objects to allow shapeshifting to occur when stimulated. Heat is the most common stimulus, most state-of-the-art prototypes are presently fabricated by a multi-material 3D printer [26,27,28]. However, due to hydrogels requiring the presence of water and often a long period of swelling, it is comparably more challenging to find suitable applications for shapeshifting hydrogels.

Another limitation while applying smart hydrogels in shapeshifting applications is that conventional hydrogels are possessed of weaker mechanical properties and easily have their mechanical properties reduced after swelling [29]. As opposed to biological tissues, such as muscles, conventional synthetic hydrogels exhibit a swelling–weakening performance caused by the dilution of the network due to the absence of biological membrane barriers that can regulate the transmembrane transport of water molecules and ions [30]. As a result, scientists have included nanoparticles [31,32,33], comonomers [34], nanobarriers [30], and crosslinkers [35,36] to improve hydrogels’ mechanical properties during synthesising. 

Furthermore, through including these materials, the swelling behaviour of smart hydrogels is also altered. For instance, by incorporating chemical crosslinkers when synthesising, the hydrogels will not only toughen them but also lower their swelling degree.

Poly (N-isopropylacrylamide) (PNIPAm) is the most used and studied thermosensitive hydrogel due to the close-to-physio LCST at 32 °C [22,37]. A second popular thermosensitive hydrogel is poly (N-vinylcaprolactam) (PNVCL), which has its LCST ranging from 30 to 32 °C [38]. Currently, PNIPAm is approved by Food Drug Administration (FDA) in drug delivery applications [39]; however, there are no FDA-approved PNVCL commercial products yet [40]. 

Implementation of PNVCL in clinical applications might seem limited presently; however, the tunability of the LCST of PNVCL has been proven practical by recent work [41]. Our team has also published the effect of applying 3D-printing and UV-curing techniques on fabricating an NVCL-based polymer using two different photoinitiators [42]. Continuous research on PNVCL will one day all add up to its successful application in numerous areas, including biomedical and shapeshifting areas. 

Therefore, in this research, our aim is to study the effects of incorporating NIPAm as comonomer along with poly (ethylene glycol) dimethacrylate (PEGDMA) as a chemical crosslinker based on different concentrations with the main monomer, NVCL, on UV-cured copolymer properties, such as swelling behaviours and LCST, for their future use in shapeshifting applications. The results show that the NVCL-based polymers are mechanically improved through the incorporation of NIPAM and PEGDMA, and the effect of such incorporation has minimal impact on the alteration of the copolymers LCST.

## 2. Results and Discussion

### 2.1. Photopolymerisation

The photopolymerisation method consumed less time and a much smaller volume of materials than most other polymerisation techniques [38]. Furthermore, the inclusion of Irgacure 2959 as a photoinitiator was essential in the photopolymerisation process, as it absorbed UV light, which used the light energy to form free radicals that subsequently initiated the polymerisation process [43]. Irgacure 2959 was chosen as it has lower cytotoxicity when compared to other photoinitiators [44]. 

PEGDMA was used as a chemical crosslinker to obtain chemical gels so that swelling studies could be performed without dissolving the gels. Unlike previous studies that applied low percentage of PEGDMA [41,42], higher percentages of PEGDMA were used to strengthen the gels and limit their maximum swelling ratio. In the shapeshifting applications, the materials would not keep up if they are too soft, and vice versa, their shape would not return to the pre-programmed shape if the shapeshift-able smart materials are too stiff [45]. 

**Figure 1:**
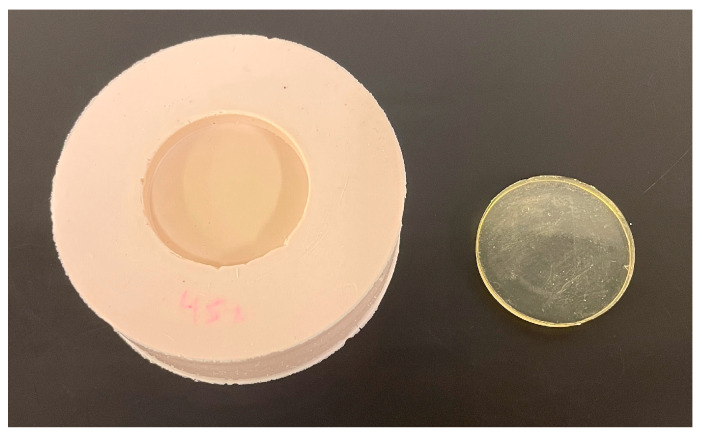
Example of a silicone mould used for the fabrication of xerogels and one of the transparent, glass-like discs prepared.

In addition, the incorporation of NIPAm in the formulations influenced the mechanical properties of cured samples due to its molecular weight and size being different from NVCL. The samples were UV-cured for 10 min on each face to ensure consistent irradiation. Silicone moulds were used to contain the prepolymerised mixtures due to their flexibility and indestructibility. Eventually, the samples cured were transparent and glass-like (Figure 1), showing amorphous features as light penetrated through the polymers instead of being absorbed and reflected. 

### 2.2. Attenuated Total Reflectance Fourier-Transform Infrared Spectroscopy

**Figure 2:**
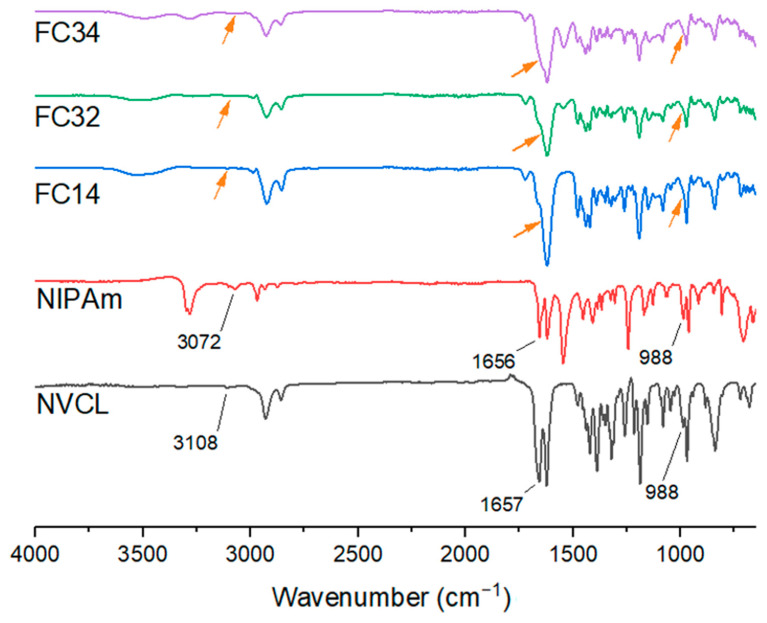
FTIR spectra showing peaks detected from monomers vanished in synthesised copolymers.

ATR-FTIR is a common technique conducted to detect molecular fingerprints and to identify the presence of any functional group of both monomers and polymers. In this study, ATR-FTIR was conducted to determine if the chemically crosslinked gels were successfully polymerized through the UV irradiation. All tested samples were oven-dried at 50 °C for 24 h prior to analysis. Figure 2 illustrates the spectra of two monomers: NVCL and NIPAm, and a few chemically crosslinked gels (FC14, FC32, and FC34). Furthermore, some important peaks from the spectra are included in Table 1. 

The amide group (C=O) for NVCL and NIPAm were found at 1623 and 1621 cm^−1^. The existence of the amide group in the chemically crosslinked gels gave a similar range of wavenumber from 1618–1622 cm^−1^. The N-H stretching (3298 and 3283 cm^−1^) and bending (1547 cm^−1^) peaks found from NIPAm were comparable to the literature [46]. Copolymers synthesised with NIPAm showed peaks due to N-H stretching and bending within these ranges. Moreover, as an important feature of monomers, the C=C bonding was detected at 1657 cm^−1^ for NVCL and 1656 cm^−1^ for NIPAm, which were both similar to the literature [46,47]. 

The disappearance of the peak at around 1655 cm^−1^ is due to the breakage of C=C bonding indicating the successful polymerisation of FC14, FC32, FC33, FC34, FC35, FC36, and FC37. The successful polymerisation was also proven by the disappearance of the peak assigned to 988 cm^−1^ in the fingerprint region that corresponded to =CH_2_ bonding for both NVCL and NIPAm, and the fading of 3108 and 3072 cm^−1^ peaks from NVCL and NIPAm that associated with the =CH bonding. Nonetheless, due to the polymerized samples absorbing moisture from the air, the peaks detected at around 3500 cm^−1^ for the chemically crosslinked gels indicated O-H stretching, which corresponded to the hydrophilic nature of the gels. The disappearance of peaks in copolymers is represented by the arrows indicated in Figure 2.

### 2.3. Phase Transition Determination for Physically Crosslinked Hydrogels

#### 2.3.1. Cloud-Point Measurements

**Figure 3:**
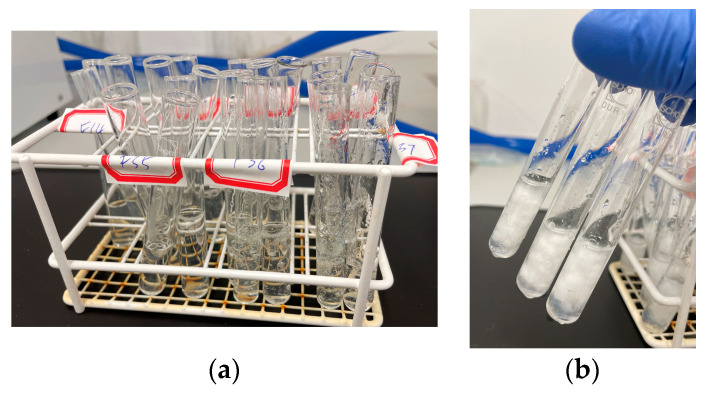
Cloud-point measurements of the aqueous solutions: (**a**) Transparent aqueous solutions at room temperature. (**b**) Upon heating, the aqueous solutions became turbid.

Cloud-point measurements were performed on the aqueous solution of physically crosslinked gels. The cloud point was the temperature where the solution started to show the first sign of turbidity. The aqueous solutions prepared were transparent at room temperature (Figure 3a); by heating, they became turbid, and polymers precipitation occurred (Figure 3b) due to stronger hydrophobic chain reactions. This is the smart feature of thermosensitive hydrogels, such as PNVCL and PNIPAm, that allows them to have different properties below and above their LCST. 

Based on the literature [47], generally 10 wt% concentration of aqueous solutions were prepared, yet in this study, 5 wt% concentration of aqueous solutions were made as the gels only fully dissolved at 5 wt%. We noticed that, by increasing the PNIPAm percentage from 10 to 30 wt%, the LCST increased from 31 to 32 °C. However, due to the similar LCST range between PNVCL [38] and PNIPAm [22], the incorporation of NIPAm proved insignificant effects on the NVCL-based copolymers’ LCST (Table 2).

#### 2.3.2. UV Spectroscopy

**Figure 4:**
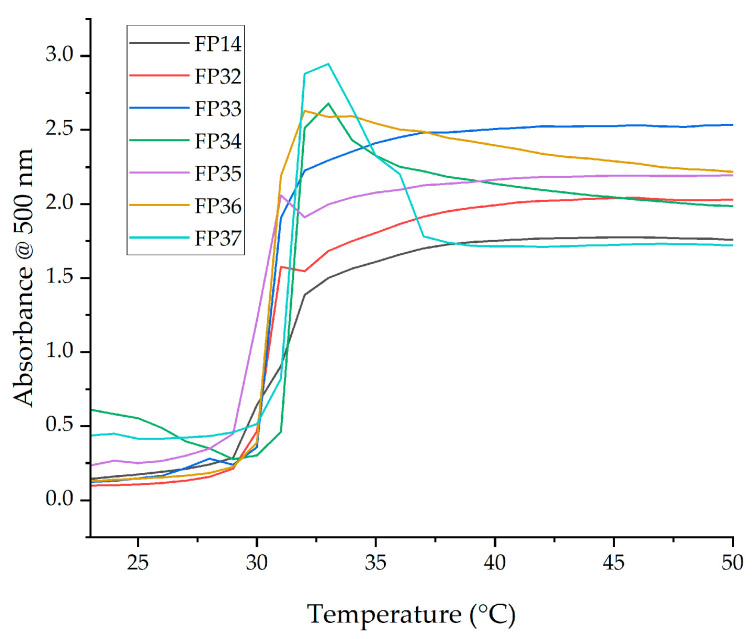
UV spectroscopy of the aqueous solutions of FP14, FP32, FP33, FP34, FP35, FP36, and FP37.

The aqueous solutions of physically crosslinked NVCL-based polymers’ LCST was also analysed using UV spectroscopy. At the set wavelength of 500 nm, the solutions absorbed light differently from a temperature range of 23–50 °C. As shown in Figure 4, the absorbance levels increased drastically starting at 30 °C. High absorbance indicated an opaque appearance of the tested samples was being detected. It is observed that with 30 wt% of NIPAm comonomer, the LCST was detected to be 1 °C higher than formulations with less NIPAm concentration (Table 2). 

In addition, the aqueous solution of formulations with higher NIPAm content showed higher viscosity than the others. A sharp decrease in absorbance was observed in samples FP34 and FP37 at a temperature of 33 °C, which could be attributed to the refraction of light caused by the presence of air bubbles formed during pipetting of aqueous samples into wells after the temperature exceeded their LCST. However, UV spectroscopy performed was to determine the LCST of the samples under the same concentration; therefore, the spectroscopy results confirmed again that the effect on the LCST through incorporating NIPAm together with NVCL was negligible. 

### 2.4. Differential Scanning Calorimetry

DSC is a technique that is used for thermal characterisation of polymeric materials. During the heating or cooling stage, the heat flow linked with material transitions as a function of temperature and time is plotted [48]. DSC analysis was conducted to determine the following: the glass transition temperature (Tg) and the LCST of the chemically crosslinked gels.

**Figure 5:**
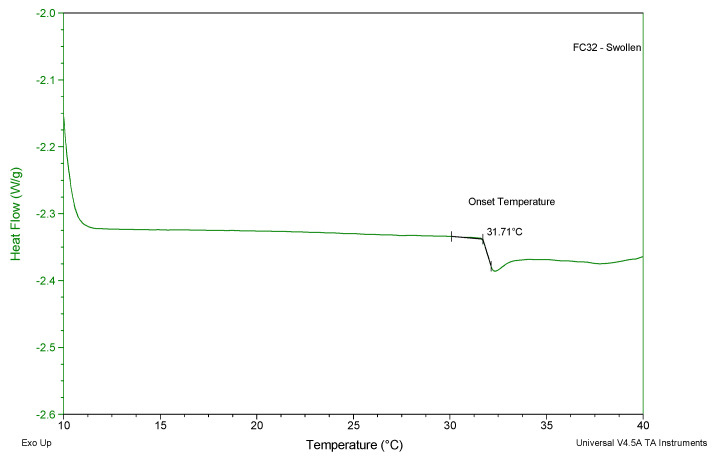
A representative DSC heating curve of a fully swollen chemically crosslinked hydrogel for analyzing its LCST.

Chemically crosslinked hydrogels were not tested for LCST using cloud-point measurements and UV spectroscopy due to the existence of covalent bonding that restricted their dissolution in water. For the determination of LCST, fully swollen chemically crosslinked hydrogels were used. A fully swollen state would improve the chance of noticing the breakage of hydrophilic bond using the DSC method. Furthermore, the heating rate was set at 1 °C/min to ensure the accuracy of the results. The LCST was found as the onset temperature of an endothermic peak (Figure 5). The LCST values ranging from 30–32 °C were observed for all chemically crosslinked gels using the DSC method. Hence, it was concluded that the incorporation of PEGDMA did not significantly affect the LCST properties of the hydrogels prepared.

**Figure 6:**
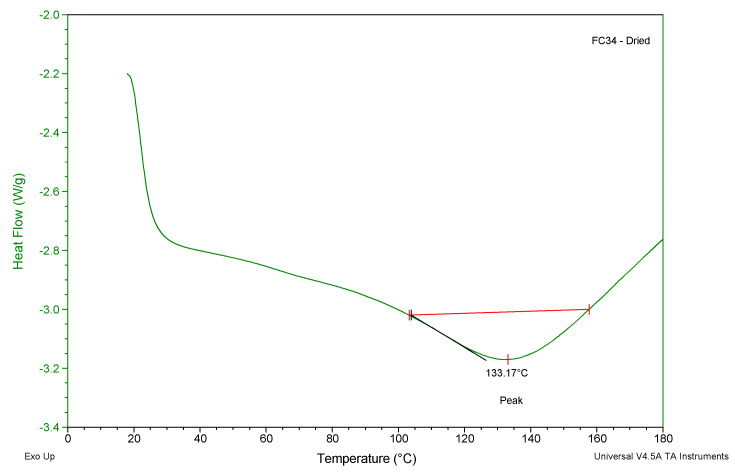
One of the dried chemically crosslinked gels analyzed for Tg using the DSC method.

Based on the literature, PNVCL has a Tg of 147 °C [49], and PNIPAm has a Tg of 133 °C [50]. The discovered peak Tg values (Figure 6) of all chemically crosslinked gels were within the range of 115–145 °C. The incorporation of three monomers: NVCL, NIPAM, and PEGDMA had a slight effect on the glass transition properties. In fact, the obtained Tg values would not matter as most hydrogels are applied in biomedicine. Therefore, the Tg results in this case are not significant for utilizing the developed formulations in the shapeshifting applications.

### 2.5. Swelling Studies

Preliminary swelling analysis was performed for all chemically crosslinked gels at room temperature. Hydrogels swell in water due to the expansion of the polymeric chains network caused by the interaction between the polymeric chains network and water molecules [51]. 

**Figure 7:**
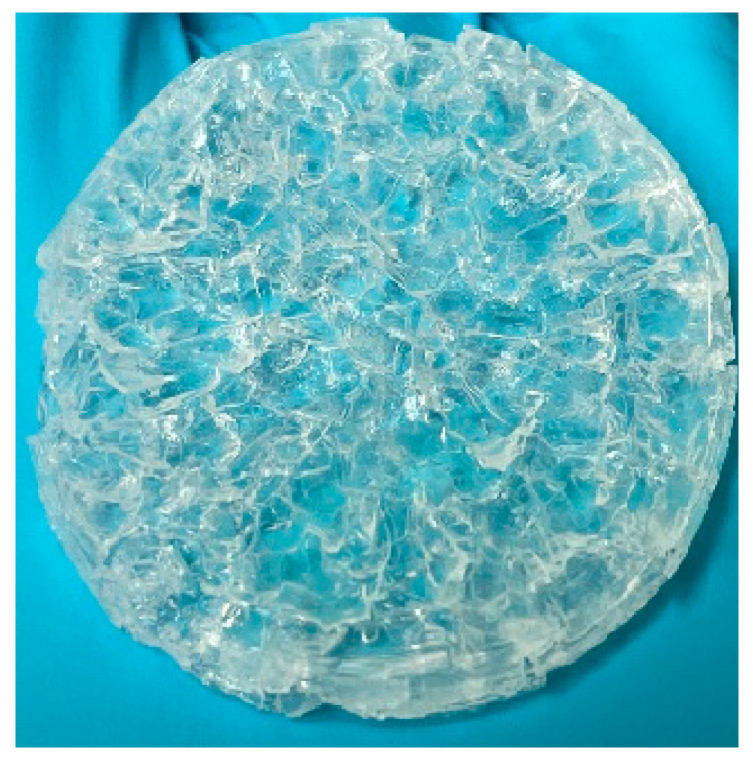
Poor surface quality of FC14 after 72 h of swelling at room temperature.

However, the mechanical properties of hydrogels degrade as the degree of swelling increases [29,52], yet the mechanical properties are essential in the shapeshifting applications. Therefore, it is crucial to develop formulations that offer satisfactory mechanical properties for making shapeshifting assemblies. First, FC14, which contained 90 wt% of NVCL and 10 wt% of PEGDMA, showed very poor surface quality after 72 h of swelling with flakes forming on the surface as shown in Figure 7. 

After being fully swollen at 20 °C for 72 h, FC14 reached a swelling ratio of 144%. However, the discs became very brittle, and the flakes formed started to peel off from the surface. This was due to the high percentage of chemical crosslinker that caused a great level of restriction to the polymer chain movement during swelling and resulted the breakage of polymer chains. Then, a great improvement in the hydrogel’s integrity was achieved for all the other chemically crosslinked hydrogels. 

**Figure 8:**
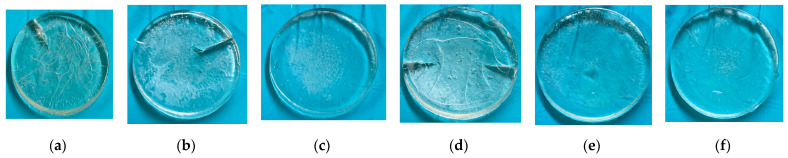
Swollen gels at equilibrium at room temperature: (**a**) FC32; (**b**) FC33; (**c**) FC34; (**d**) FC35; (**e**) FC36; and (**f**) FC37.

As can be seen in Figure 8, flakes were no longer formed on the disc surface although minor cracks occurred on some of the discs. The incorporation of NIPAm as a comonomer has a positive impact on the structural integrity of NVCL-based hydrogel when a high percentage of crosslinker was used. Accordingly, it was observed that higher concentrations of NIPAm yielded better hydrogel’s integrity. For instance, FC34 (30 wt% of NIPAm), FC36 (20 wt% of NIPAm), and FC37 (30 wt% of NIPAm) were the three formulations that showed perfect appearance after swelling to equilibrium at room temperature. 

**Figure 9:**
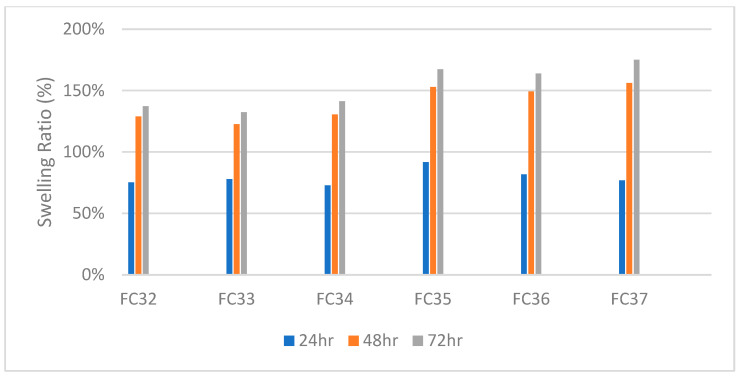
Swelling ratio (%) of formulations FC32–FC37.

While FC33 contained 20 wt% of NIPAm like FC36, the crack may be due to higher crosslinker percentage that caused more restriction to the polymer chain movement. Moreover, the swelling ratios of formulations FC32–FC37 are plotted in Figure 9. It is worth mentioning that FC32–FC34 that contained 10 wt% of crosslinker swelled about 25% less than FC35–FC37 that contained 7.5 wt% of crosslinker. The crosslinking density affecting swelling capacity of hydrogels inversely matches with the literature [53]. Eventually, FC34, FC36, and FC37 were selected for pulsatile swelling studies.

#### Pulsatile Swelling Studies

To unearth more about the swelling ability of the selected formulations, pulsatile swelling studies were performed. At two different temperatures (20 and 50 °C), swelling of FC34, FC36, and FC37 samples in distilled water was performed until equilibria weight was achieved. The swelling begun at 20 °C until equilibrium then continued at 50 °C in an oven, and the entire process was repeated twice more. It was observed that either formulation reached their maximum swelling ratio at 72 h, and it took less than 24 h for all discs to reach equilibria weight after being placed in the oven. In addition, in the second cycle of swelling the discs spent merely 24 h (instead of 72 h in the first cycle) to reach to their maximum swelling ratio. 

**Figure 10:**
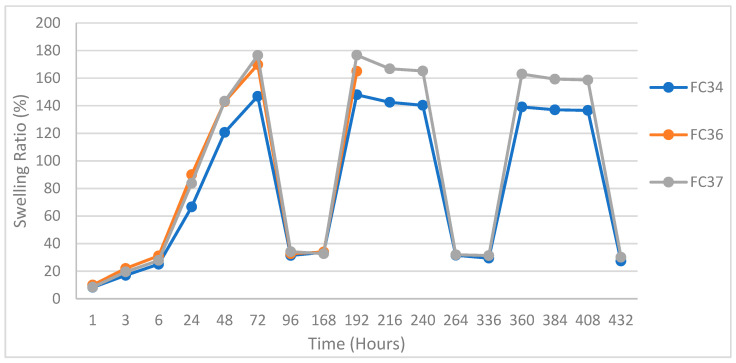
Pulsatile swelling behaviours of FC34, FC36, and FC37 at 20 and 50 °C. First cycle: 20 °C in the beginning, 50 °C from 72 h; second cycle: 20 °C from 168 h, 50 °C from 240 h; and third cycle: 20 °C from 336 h and 50 °C from 408 h.

**Figure 11:**
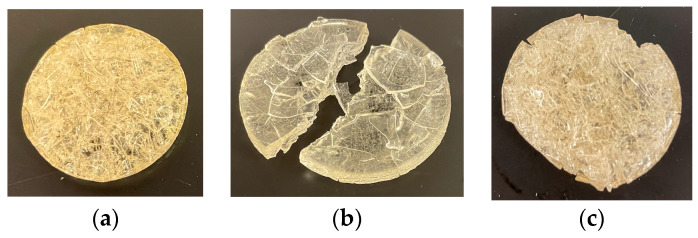
Chemically crosslinked gels tested from pulsatile swelling studies: (**a**) Dried FC34 after three cycles; (**b**) broken FC36 at 192 h; and (**c**) dried, partially broken FC37 after three cycles.

**Figure 12:**
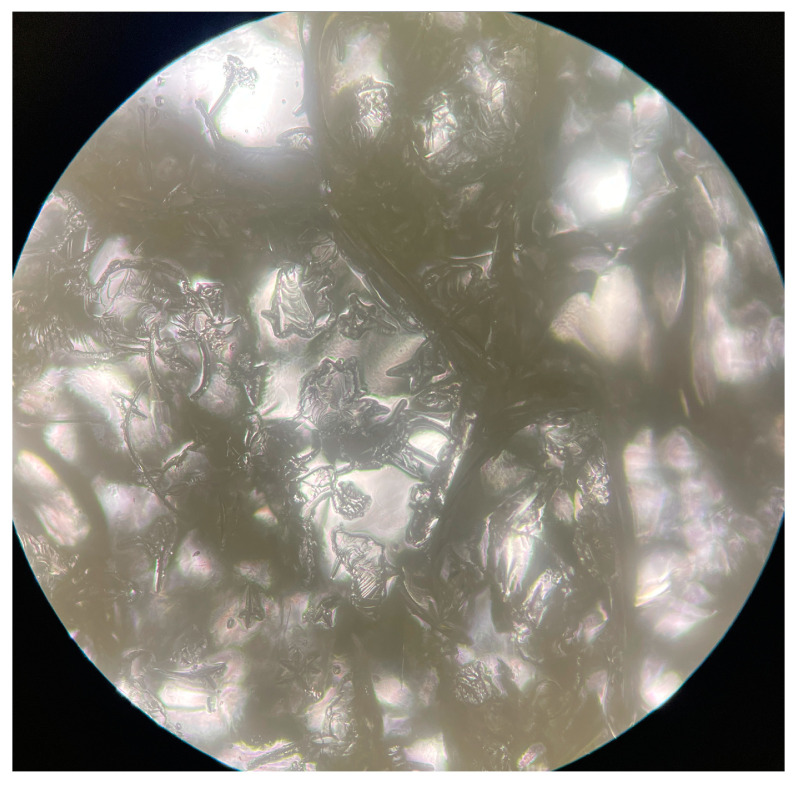
Microscopic view of dried FC34 disc after three cycles of pulsatile swelling (Olympus CX23, magnification 40×).

The pulsatile swelling continued until all three cycles were completed, and the result is plotted in Figure 10. Both FC34 and FC37 completed three cycles of pulsatile swelling, yet FC36 broke into halves in second cycle (Figure 11b). This could be due to the lower percentage of NIPAm (20 wt%) when compared to both FC34 and FC37, which comprised 30 wt% of NIPAm. Furthermore, after three complete cycles, the tested FC34 and FC37 discs were fully dried in the oven at 50 °C. FC37 discs were partially broken (Figure 11c) while FC34 discs still maintained their structural integrity (Figure 11a) after drying, although, cracks were observed throughout the discs (Figure 12). 

The phenomena of crack development and breakage of discs were due to the sudden formation of internal stresses, while the temperature rose to above the LCST that caused the polymer chains shrink and expel water molecules through hydrophobic segments [54]. The FC34 discs preserving their structural integrity after three cycles of pulsatile swelling may be due to less internal stresses generated within their polymer network when compared to FC36 and FC37. 

Such outcome also indicates that NVCL formulation with high percentage of PEGDMA requires another comonomer like NIPAm to improve their structural integrity. FC34, a chemically crosslinked NVCL/NIPAm hydrogel with the best swelling integrity was successfully developed. Future improvement is required to prevent the formation of cracks within the gel during pulsatile swelling for prolonging their application lifetime in the thermosensitive area. 

### 2.6. Rheological Analysis

It is challenging when it comes to examining the mechanical properties of hydrogels. Hydrogels generally become much weaker after swelling and the conventional instruments used for analysing thermoplastics mechanical properties are not all applicable for analysing the same for hydrogels. For instance, most conventional mechanical testing instruments expect samples to be clamped on the fixture while carrying out the analysis, while this requirement would have possibly destroyed the hydrogels. 

Therefore, to compare the mechanical strength of each formulation by causing minimal destruction to the hydrogels, a rheometer equipped with parallel plate was used. In general, storage modulus (G′) is the indication of the energy stored in the elastic structure of the sample. In this case, only G′ tested for each sample were compared as it brings the most interest about the effect of including the crosslinker and comonomer on the hydrogel’s stiffness. 

**Figure 13:**
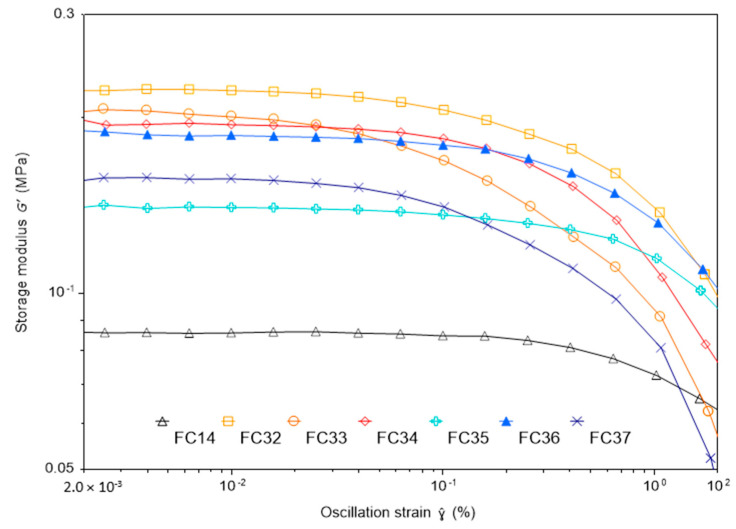
A plot of storage modulus examined for each of the chemically crosslinked hydrogels.

Distinct from the usual testing techniques for hydrogels in liquid form [55], a constant normal force of 3 +/− 0.3 N was applied to the chemically crosslinked hydrogels due to different swelling ratios that resulted different thicknesses of the samples. As shown in Figure 13, the incorporation of NIPAm as a comonomer has increased the G′ of FC14 (90 wt% of NVCL with 10 wt% of PEGDMA) from about 85 to above 150 kPa. Another point worth mentioning is that copolymer formulations having a higher percentage of PEGDMA (10 wt%) showed comparably higher G′ at above 200 kPa in the linear viscoelastic region, while copolymer formulations having lower percentage of PEGDMA (7.5 wt%) showed lower G′ at below 200 kPa in their linear viscoelastic region. 

The phenomenon is due to more covalent bonds formed between the polymer chains when higher PEGDMA concentration is incorporated, which, in turn, enhances the stiffness of the swollen samples. The outcome of rheological analysis proved that the mechanical performance of NVCL-based hydrogels was improved overall by including NIPAm as a comonomer and PEGDMA as a crosslinker.

## 3. Conclusions

By using the photopolymerisation technique, the fabrication of NVCL-NIPAm copolymers chemically crosslinked with PEGDMA was successful, which was proven by FTIR. Cloud-point analysis and UV spectroscopy were performed on an aqueous solution of the physically crosslinked copolymer, and the authors confirmed that NIPAm has a negligible effect in tilting the copolymer’s LCST due to its similar LCST range with NVCL. Differential scanning calorimetry was used to further conclude that PEGDMA did not significantly affect the LCST. 

The glass transition temperature detected for the chemically crosslinked copolymers lay within the irrelevant range for their applications in biomedicine. FC34, FC36, and FC37 showed excellent structural integrity after being fully swollen at room temperature. However, only FC34 and FC37 completed three cycles of pulsatile swelling at both 20 and 50 °C. The pulsatile swelling revealed that FC34 had the best structural integrity post-process, although the formation of cracks was noticed. Finally, the rheological analysis discovered that the incorporation of NIPAm and PEGDMA significantly improved the hydrogels mechanical performance in their viscoelastic region. 

While further improvements to the best formulation are to be made, FC34 demonstrated its ability to swell at temperatures below and above its LCST repeatedly for three cycles. This creates great potential for FC34 to be used as a thermosensitive bio-actuator. Future work that includes the incorporation of nanoparticles with F34 will be pursued for the prolonged application lifetime in thermosensitive areas. Since the applications of hydrogels in biomedicine usually involve environmental factors, such as pH, ions, and blood, the performances and characterizations of the developed formulation must be verified under the applying conditions when a specific prototype is fabricated.

## 4. Materials and Method

### 4.1. Materials

N-vinylcaprolactam (NVCL) (Mw = 139.19 g/mol) was obtained from Sigma Aldrich Ireland (Dublin, Ireland). Another monomer, N-Isopropylacrylamide (NIPAm) (Mw = 113.16 g/mol) was obtained from TCI Europe (Paris, France). The chemical crosslinker, poly (ethylene glycol) dimethacrylate (PEGDMA) (Mw = 550 g/mol) was provided by Sigma Aldrich Ireland (Dublin, Ireland). The photoinitiator, 4-(2hydroxyethoxy) phenyl-(2-hydroxy-2-propyl) ketone (Igracure^®^ 2959 Ciba Corp, New York, NY, USA) was obtained from Ciba Specialty Chemicals (Basel, Switzerland). Table 3 below illustrates the chemical structure of each material used.

### 4.2. Synthesis of NVCL-Based Copolymers

The fabrication of the NVCL-based copolymers was performed through free-radical polymerisation using UV irradiation. A UV-irradiation chamber (Dr. Gröbel UV-Elektronik GmbH, Ettlingen, Germany) that contained 20 UV tubes was utilised. This UV-irradiation chamber provides a spectral range of UVA between 315–400 nm at an average intensity of 10–13.5 mW/cm^2^. Seven different formulations that contained NVCL, NIPAm, PEGDMA, and Iragacure 2959, as shown in Table 4, were pre-mixed based on the table-listed percentages in individual beakers. Subsequently, the mixtures were stirred using a magnetic stirrer at 150 rpm and 50 °C for 20 min until homogeneous. The pre-polymerized solutions were then pipetted into several disc impression-containing silicone moulds. The circular disc impressions had a thickness of 4 mm and a diameter of 30 mm. Photopolymerisation was then conducted for 10 min on each face of the circular discs. Cured samples were dried in a vacuum oven at 50 °C for 24 h. To complete the characterisations of all formulations, physically crosslinked versions of each formulation were fabricated following the same method according to Table 5.

### 4.3. Attenuated Total Reflectance Fourier Transform Infrared Spectroscopy

Attenuated total reflectance Fourier transform infrared spectroscopy (ATR-FTIR) was performed on a Perkin Elmer Spectrum One FT-IR Spectrometer (Perkin Elmer, Waltham, MA, USA). equipped with a universal ATR sampling accessory. All examinations were conducted at room temperature in the spectral range of 4000–650 cm^−1^, utilizing a four-scan-per-sample cycle under a constant universal compression force of 75 N. The collected data was analysed using the Spectrum software (2022.1.0, Tokyo, Japan).

### 4.4. Preparation of Aqueous Solutions

The fabricated physically crosslinked gels (Table 5) were dissolved in distilled water with a ratio of 0.5:9.5 (gel: distilled water) to form aqueous solutions of 5 wt% hydrogel concentration. The mixtures were kept in capped mechanical flasks at room temperature until the hydrogels were completely dissolved.

#### 4.4.1. Cloud-Point Analysis

Cloud-point measurement was used to discover the phase transition temperature of the dissolved hydrogels. The aqueous solutions that were previously prepared were pipetted into separate test tubes, and the test tubes were immersed in a Memmert water bath system. A thermometer with accuracy of +/− 0.2 °C was placed in the water bath during the analysis for temperature monitoring. Starting from 20 °C, the temperature was slowly increased for 1 °C per 2 min to ensure consistency of temperature change. The temperature was recorded when the solution started to show turbidity until the degree of turbidity became constant. 

#### 4.4.2. UV Spectroscopy

UV spectroscopy was another method used to discover the phase transition temperature of the fabricated aqueous physically crosslinked formulations. A Synergy HT BioTek plate reader fitted with a heating system was operated. The spectroscopy was set at 500 nm for optical absorbance from 23–50 °C. For every 1 °C, the 96-well plate was allowed to equilibrate for 20 min before the measurement of absorbance.

### 4.5. Differential Scanning Calorimetry

The chemically crosslinked xerogels were analysed using a Pyris 6 DSC (Perkin Elmer, Waltham, MA, USA). An empty aluminium sample pan sealed with a lid was used as a reference sample for the test. The gel samples were weighed between 8–10 mg using a Sartorius scale with 0.01 mg resolution. Both swollen and dry gels for each chemically crosslinked formulation (Table 2) were tested based on different parameters. The swollen gels were tested from 10–60 °C at 1 °C/min. Moreover, the dried gels were tested from 20–200 °C at 10 °C/min. A constant nitrogen gas flow at 20 mL/min was applied while performing all tests. The obtained curves were plotted as a function of heat flow (W/g) against temperature (°C) and further processed using the TA Universal Analysis software (2000 Manual, New Castle, DE, USA).

### 4.6. Swelling Studies

The swelling studies were performed in triplicate by placing the chemically crosslinked gels (Table 2) into the Thermo Scientific™ Sterilin™ (Waltham, MA, USA) petri dishes (diameter = 90 mm, height = 15.9 mm), which were later fully filled with distilled water. All the analyzed samples had dry weight measured at 2.5 +/− 0.25 g. The swelling studies were performed at room temperature. Intermittently, the gel samples were removed from the petri dishes and blotted free of surface water using filter papers, and the swollen weights were measured using a Sartorius scale at room temperature. 

The gel samples were then resubmerged in distilled water until the next measurement was taken, and the cycle was repeated until the constant weight of the gel samples was achieved. The swelling percentage of the samples was calculated using Equation (1), where W_t_ represents the weight of the swollen gel at a predetermined time and W_d_ represents the dry weight of the gel.

Swelling Ratio (%) = (W_t_ − W_d_)/W_d_ × 100% (1)

#### Pulsatile Swelling Studies

Based on the result from swelling studies, candidates showing the best integrity at a fully swollen state were chosen for further swelling back and forth repeatedly at both 20 °C (room temperature) and 50 °C. A cycle consisted of an equilibria weight achieved at both temperatures, and three cycles were performed for each selected formulation. The swelling percentages were calculated using Equation (1), and the results are plotted in a graph (Figure 10).

### 4.7. Rheological Analysis

The amplitude sweep was conducted on fully swollen chemically crosslinked hydrogels as a comparative test for their mechanical strength. A Discovery HR30 rheometer (TA Instruments, New Castle, DE, USA) installed with a stainless-steel Peltier plate, including temperature control was utilised for the analysis. A 20 mm diameter stainless-steel parallel plate was used for testing all samples. The amplitude sweep was conducted from 0.0001 to 10% of strain at 1 Hz frequency. A constant normal force of 3 +/− 0.3 N was applied to all tests at a set temperature of 20 °C. The storage moduli (G′) were compared using a graph.

## Figures and Tables

**Table 1:** Some important FTIR bands for monomers and synthesised copolymers.

Functional Group	Wavenumber (cm^−1^)								
	NVCL	NIPAm	FC14	FC32	FC33	FC34	FC35	FC36	FC37
N–H	-	3298, 3283, 1547	-	3288, 1542	3288, 1541	3288, 1542	3278, 1542	3279, 1542	3278, 1542
C–H	2930, 2859	2969, 2933	2925, 2856	2926, 2857	2922, 2864	2928, 2859	2926, 2856	2926, 2857	2928, 2867
C=O	1623	1621	1622	1619	1620	1622	1622	1620	1618
C=C	1657	1656	-	-	-	-	-	-	-
=CH	3108	3072	-	-	-	-	-	-	-
=CH_2_	988	988	-	-	-	-	-	-	-
O–H	-	-	3526	3505	3505	3504	3497	3497	3504

**Table 2:** Comparison table illustrating the LCST values obtained from cloud-point analysis and UV spectroscopy.

Formulation	Cloud Point (°C)	UV Spectroscopy (°C)
FP14	30	30
FP32	31	30
FP33	31	30
FP34	32	31
FP35	31	30
FP36	31	30
FP37	32	31

**Table 3:** Materials used and their chemical structure.

Materials	Chemical Structures
N-Vinylcaprolatam (NVCL)	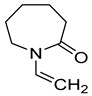
N-Isopropylacrylamide (NIPAm)	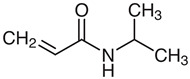
Poly (ethylene glycol) dimethacrylate (PEGDMA)	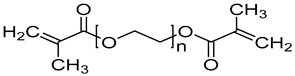
4-(2Hydroxyethoxy) phenyl-(2-hydroxy-2-propyl) ketone (Irgacure 2959)	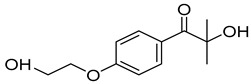

**Table 4:** Compositions of chemically crosslinked xerogels.

	Monomers		Crosslinker	Photoinitiator	
Formulation	NVCL (wt%)	NIPAm (wt%)	PEGDMA (wt%)	Irgacure 2959 (wt%)	Total
FC14	89.91	0.00	9.99	0.10	100
FC32	79.92	9.99	9.99	0.10	100
FC33	69.93	19.98	9.99	0.10	100
FC34	59.94	29.97	9.99	0.10	100
FC35	82.42	9.99	7.49	0.10	100
FC36	72.43	19.98	7.49	0.10	100
FC37	62.44	29.97	7.49	0.10	100

**Table 5:** Compositions of physically crosslinked xerogels.

	Monomers		Crosslinker	Photoinitiator	
Formulation	NVCL (wt%)	NIPAm (wt%)	PEGDMA (wt%)	Irgacure 2959 (wt%)	Total
FP14	99.90	0.00	0	0.10	100
FP32	88.80	11.10	0	0.10	100
FP33	77.70	22.20	0	0.10	100
FP34	66.60	33.30	0	0.10	100
FP35	89.10	10.80	0	0.10	100
FP36	78.30	21.60	0	0.10	100
FP37	67.50	32.40	0	0.10	100
